# Dr. Elbert Augustus Ainsworth

**Published:** 1916-12

**Authors:** 


**Dr. Elbert Augustus Ainsworth**, Syracuse 1874, died at his home in West Union, Ia., Aug. 31, aged 67. He was City Health Officer, a member of the board of education and surgeon to the C. M. & St. P. R. R.

